# 5-Androstene-3β,7β,17β-triol (β-AET) Slows Thermal Injury Induced Osteopenia in Mice: Relation to Aging and Osteoporosis

**DOI:** 10.1371/journal.pone.0013566

**Published:** 2010-10-21

**Authors:** Ajay K. Malik, Sophia Khaldoyanidi, Dominick L. Auci, Scott C. Miller, Clarence N. Ahlem, Christopher L. Reading, Theodore Page, James M. Frincke

**Affiliations:** 1 Harbor Biosciences, Inc., San Diego, California, United States of America; 2 Torrey Pines Institute for Molecular Studies, San Diego, California, United States of America; 3 Radiobiology Division, University of Utah, Salt Lake City, Utah, United States of America; Universidad Europea de Madrid, Spain

## Abstract

5-androstene-3β,7β,17β-triol (β-AET), an active metabolite of dehydroepiandrosterone (DHEA), reversed glucocorticoid (GC)-induced suppression of IL-6, IL-8 and osteoprotegerin production by human osteoblast-like MG-63 cells and promoted osteoblast differentiation of human mesenchymal stem cells (MSCs). In a murine thermal injury model that includes glucocorticoid-induced osteopenia, β-AET significantly (*p*<0.05) preserved bone mineral content, restored whole body bone mineral content and endochondral growth, suggesting reversal of GC-mediated decreases in chondrocyte proliferation, maturation and osteogenesis in the growth plate. In men and women, levels of β-AET decline with age, consistent with a role for β-AET relevant to diseases associated with aging. β-AET, related compounds or synthetic derivatives may be part of effective therapeutic strategies to accelerate tissue regeneration and prevent or treat diseases associated with aging such as osteoporosis.

## Introduction

Dehydroepiandrosterone (DHEA) and its sulfate are the major circulating adrenal steroids in humans. Serum levels peak in young adults, but then steadily decline with age, falling over 80% by age 70. Thus, DHEA is thought to be a good biomarker for aging, although its biological role remains poorly understood. DHEA demonstrates a plethora of anti-aging properties in rodents, including anti-inflammatory [1], [2], [3], anti-obesity [4], and immune enhancing [5] activities. In rodents, DHEA opposes certain activities of endogenous glucocorticoids (GC) such as osteoporosis [6], [7], [8], and improves osteoblast growth and bone tissue morphometry [9]. As the literature grew, DHEA became widely used as an anti-aging, anti-stress dietary supplement and was considered a promising agent to treat osteoporosis. Despite promising results in rodents, DHEA provided only modest improvement in bone mineral density (BMD) in patients treated with GC, and elicited unwanted androgenic side effects [10], [11], [12], [13].

Such widely different outcomes in rodents and humans have been referred to as ‘the DHEA conundrum’. With respect to bone preservation in rodents, DHEA was thought to act directly *via* a DHEA-specific receptor [9], while this same activity (bone preservation) appears to require conversion to sex steroids in humans [14]. Species-specific differences in the adrenal metabolome exist and may play a significant role in the lack of translation between species. DHEA is abundant in human circulation, but exogenous DHEA has poor oral bioavailability and is primarily metabolized into sex steroids, which yield the corresponding side effects rather than the desired benefits [15], [16]. In contrast, DHEA levels do not existent in rodents [17], and exogenous DHEA is efficiently converted into highly oxidized metabolites [18]. Rodent metabolites include a number of androstene and androstane derivatives that result from additional ring oxidation at carbon 7 and 16 [18], [19], [20], [21], [22], [23]. Many of the functions initially attributed to DHEA from observations in rodents are now thought to be properties of these oxygenated metabolites.

Oxidation *via* the action of the enzyme CYP7B leads to the 7-hydroxy derivatives of C-19 steroids [24], [25], which are collectively present in low nanomolar concentrations in human circulation and are not readily metabolized to potent androgens or estrogens. Androstene-3β,7β,17β-triol (β-AET) represents a key naturally occurring 7-hydroxy DHEA metabolite that possesses some of the anti-inflammatory and GC-opposing activities that have been attributed to DHEA, but with greater apparent potency [23], [26], [27], [28]. Loria and colleagues showed that co-culturing GC-treated Concanavalin A-induced splenocyte with β-AET antagonized the suppression of IL-2 and IL-3 production and cell proliferation [29], [30]. β-AET was also found to regulate macrophage cytokine secretion and to again oppose GC effects [31]. Co-culture with β-AET countered the suppressive effect of GC on LPS-induced TNFα and IL-1β secretion. In rodents, they also showed that DHEA and β-AET countered the GC-induced suppression of IL-4 and IgE production, thereby limiting a GC-induced Th2 shift [32]. These observations suggested that β-AET has an intrinsic role in the counter regulation of GC.

In the present studies, we probed the GC modulating function of β-AET in the context of its potential GC counter-regulatory role in bone biology. We tested if β-AET could effect human bone marrow derived MSC fate decisions towards the osteoblast lineage and drive the differentiation and functional activity of human osteoblast-like MG-63 cells. In rodents, we expanded these studies using a mouse model of thermal injury that includes an acute phase GC-induced osteopenia (GIOP) and tested whether β-AET could spare bone loss in response to thermal injury [33], [34], [35]. In humans, we correlated falling plasma levels of β-AET with aging. Our observations are consistent with a GC- counter regulatory role for β-AET relevant to bone biology and advancing age. Treatment with β-AET, related compounds or synthetic derivatives may be part of an effective therapeutic strategy to promote tissue regeneration, prevent or delay the onset of osteoporosis associated with trauma or advancing age.

## Materials and Methods

### Test Article

5-Androstene-3β,7β,17β-triol (β-AET) was produced by Harbor Biosciences (San Diego, California). *In vivo* studies used HERF405 vehicle comprised of 0.1% carboxymethylcellulose, 2% polysorbate 80 and 0.1% metabisulfite in phosphate-buffered saline, pH 7.4.

### *In vitro* studies

#### MG-63 cells

Human sarcoma cell line MG-63 (ATCC: CRL-1427) was maintained in Minimal Essential Medium (Eagle's MEM) supplemented with 0.1 mM non-essential amino acids, 1 mM sodium pyruvate, 2 mM **L**-glutamine, 100 U/mL penicillin, 100 µg/mL streptomycin and 10% heat-inactivated Fetal Bovine Serum (FBS). During experimentation, phenol red-free MEM medium containing 10% charcoal-dextran adsorbed FBS was used (assay medium). Typically 0.75×10^6^ cells were plated per well in a 6-well plate in 1 mL regular medium for 16-18 hours. The next morning, fresh assay medium was added along with β-AET (2 µL in DMSO added to 1 mL). After one hour of pre-incubation with β-AETa.k.a HE2200; prepared by Harbor Biosciences), 50 nM dexamethasone (Dex), previously determined to be approximately IC_50_ concentration with respect to inhibition of cytokines) was added (in 2 µL DMSO, final concentration 0.4%). After 7 h later, levels of IL-6, IL-8 or osteoprotegrin (OPG) were measured in the culture medium using Quantikine ELISA kits (R & D Systems, Inc., Minneapolis, MN).

#### Human MSC

Mesenchymal stem cells were derived from human bone marrow obtained from healthy volunteers (Clonetics, Inc., San Diego, CA). Bone marrow resident mesenchymal stem cells (MSCs) were obtained as previously described [36]. The resulting CD45^−^ Glycophorin A^−^ cell population was expanded in culture in the presence of Epidermal Growth Factor and Platelet-Derived Growth Factor BB (Millipore, Billerica, MA). The resulting adherent cell population was released with trypsin and differentiated into the pre-osteoblast population using 10 mM β-glycerophosphate, 10 nM Dex and 50 nM ascorbic acid phosphate as previously described [37]. Subcultures derived from trypsinized adherent cells were supplemented with either 0, 0.1, 1 or 10 µM β-AET and cultured for 15 days. The adherent population was once again trypsinized and then enumerated by flow cytometry. The region comprising differentiated mesenchymal stem cells including preosteoblast cells was identified using forward scatter and side scatter characteristics and defined as a region of interest R1. The average percent of cells expressing the preosteoblast integrins osteopontin (OP) and osteonectin (ON) were measured by FACScan analysis by flow cytometry.

#### Flow cytometry

OP- and ON-specific rabbit anti-human antibodies were from Calbiochem (San Diego, California). For flow cytometry, 5×10^5^ cells were stained according to standard procedures. Briefly, the cells were incubated with a primary antibody (10 µg/ml) for 30 min at 4°C. Control cells were incubated with isotype-matched IgG (Strategic Biosolutions, DE). After washing with FACS buffer (2%FCS, 0.1% BSA, 0.01%NaN_3_ in PBS), the cells were incubated with a goat-anti-rabbit FITC-conjugated secondary antibody (Santa Cruz, CA). Fluorescence intensity was analyzed on a FACScan (Becton Dickinson, San Jose, CA) according to standard procedures.

### Mouse osteoporosis model

#### Animals and treatment

Male BALB/c mice (12–14 weeks old, ∼25 g) were obtained from Charles River Labs, Wilmington, MA. All groups contained 10 animals. Mice were subjected to 20% total body surface area (TBSA) burn by exposing shaved dorsal skin to a 70°C water bath for seven seconds as described [35]. β-AET (or vehicle alone) was injected (50 µL) subcutaneously (sc) at a dose of 0.6 or 1.2 mg/mouse (approximately 25 and 50 mg/kg, respectively) immediately after thermal trauma; a second dose was administered 48 hours later and then 3 times/week for 4 weeks. The ‘sham’ group was treated with vehicle (50 µL) but received no TBSA. Animals used for baseline measurements received neither TBSA nor any treatment. The fluorochrome bone marker calcein (Sigma, St. Louis, MO), used for bone formation measurements, was administered seven and two days before necropsy as previously described [35]. Animal housing and experimental manipulations were done at The University of Utah Radiobiology facilities and were approved by the Institutional Animal Care and Use Committee.

#### Bone weights and chemistry

Right femurs of the mice were cleaned of adherent tissues and stored frozen in moist saline wrapping. These were later weighed when wet (wet weight), dried with acetone & ethyl ether and were re- weighed (dry weight). The dried femurs were then ashed in a muffle furnace (600°C for 12 hours) and weighed (ash weight). The ash to dry weight ratios are considered an indicator of the amount of inorganic and organic composition, respectively.

#### Bone mineral content and density

Bone mineral content (BMC) and bone mineral density (BMD) were determined by peripheral dual energy x-ray absorptiometry (DXA) using a Norland dual x-ray absorptiometer at baseline (two days prior to thermal trauma) and on the day of necropsy [35]. The scanned regions included the entire mouse (excluding the tail), the hind limbs (femur and tibia) and the lumbar spine. Additionally, the femurs and tibiae were scanned *ex vivo*. BMD is expressed as g/cm^2^.BMC is expressed in g.

#### Bone morphometry and histomorphometry

The left femur, right and left proximal tibiae, right and left tibio-fibular junctions and lumbar vertebrae of mice were processed for bone histomorphometry. The left femur (mid-shaft region) and right and left tibio-fibular junctions were sawed in cross sections. Right and left proximal tibiae were sectioned in the frontal plane. The bones were cut using IsoMet® precision bone saw (Buehler Ltd., Lake Bluff, IL), and sections were mounted on plastic slides, ground and polished for histology and histomorphometry. The static measurements were collected using a digitizer with a Nikon microscope camera lucida system with a microprocessor and customized histomorphometry software (KSS Scientific Consultants, Magna, UT). Additionally, digital images of the cross-sections were captured and structural data was obtained using the public domain NIH image analysis software.

Tibio-fibular junction region and the mid-diaphyseal shaft of the left femur were used for cortical bone histomorphometric analysis and the secondary spongiosa of the proximal tibial metaphysis for acquiring the cancellous bone parameters. The cortical bone static measures included cortical bone area, marrow area (medullary cavity), average cortical width (from periosteal to endocortical surface in cross-sections), and minimal cortical width. The “dynamic” cortical bone growth measurements were collected using the calcein fluoromarker at the periosteal and endocortical single- and double-labeled surfaces (% sLS and %dLS, respectively). Calculated periosteal and endocortical parameters were mineralizing surface (% MS, which was calculated as % dLS plus one-half of % sLS), mineral apposition rate (MAR), and surface- and volume-referent bone formation rate (sBFR and vBFR, respectively; calculated from % MS and % dLS. Cortical MAR was not corrected for obliquity. The endocortical eroded surface, is the surface that has resorption pits (Howship's lacunae).The cancellous bone parameters were % bone (percent of metaphyseal area adjoining to the growth plate), perimeter to area ratio (is an indicator of trabecular thickness), % sLS, % dLS, % MS, MAR (corrected for section obliquity by multiplying the measured rate by π/4), and surface-referent BFR (which is the BFR [active surface times MAR] normalized to the bone area).

#### Longitudinal bone growth (endochondral osteogenesis)

Endochondral growth, a function of proliferation and maturation of the growth plate cartilage, was measured at the proximal tibial metaphysis of mice and is expressed as growth per day. This was measured using the fluorochrome bone markers and represents the growth rate near the end of the study.

#### Statistical analysis

One-way ANOVA with Tukey's multiple comparison (using GraphPad Prism software) was used for statistical analyses of the in vitro data. Data was plotted as means ± SEM, unless indicated otherwise in legends. For mouse studies, ANOVA with Fisher's Protected Least Significant Difference (PLSD) was used, p<0.05 was considered to be significant.

### Human studies

#### Subjects

Plasma samples were obtained from 252 volunteers (102 males and 150 females; ages 20–80). Written informed consent was obtained from all patients as approved by the institutional review boards (Western IRB, Seattle, WA and RCRC IRB, Austin TX). Studies were conducted in accordance with the Declaration of the Helsinki and International Conference on Harmonisation/WHO Good Clinical Practice standards.

#### βAET in human subjects

The concentration of β-AET was measured in plasma by LC-MS/MS. Plasma samples (0.2 mL) were spiked with D3-β-AET as an internal standard (1 ng/mL), and steroids were extracted with 10 mL ethyl acetate, and evaporated under nitrogen. The dried residue was derivatized with 0.5 mL of 50 mg/mL nicotinyl chloride in anhydrous pyridine for 1 hour at 80°C, cooled to room temperature, and quenched with 1 mL 5% sodium bicarbonate. The derivatized steroids were extracted with 10 mL methyl-tert-butyl ether, dried under nitrogen, dissolved in HPLC mobile phase, and analyzed by reverse phase LC-MS/MS. Tri-nicotinyl-β-AET was identified by its retention time and 622>376 amu transition. A standard curve from 10–1000 pg/mL in water was used for quantification after demonstrating equivalence to the method of standard addition.

#### Statistical Analysis

Data were analyzed using Graphpad Prism 4 software (Prism, San Diego, CA).

## Results

### β-AET opposes GC-induced effects on MG-63 osteoblast-like cell line *in vitro*

**Figure 1:**
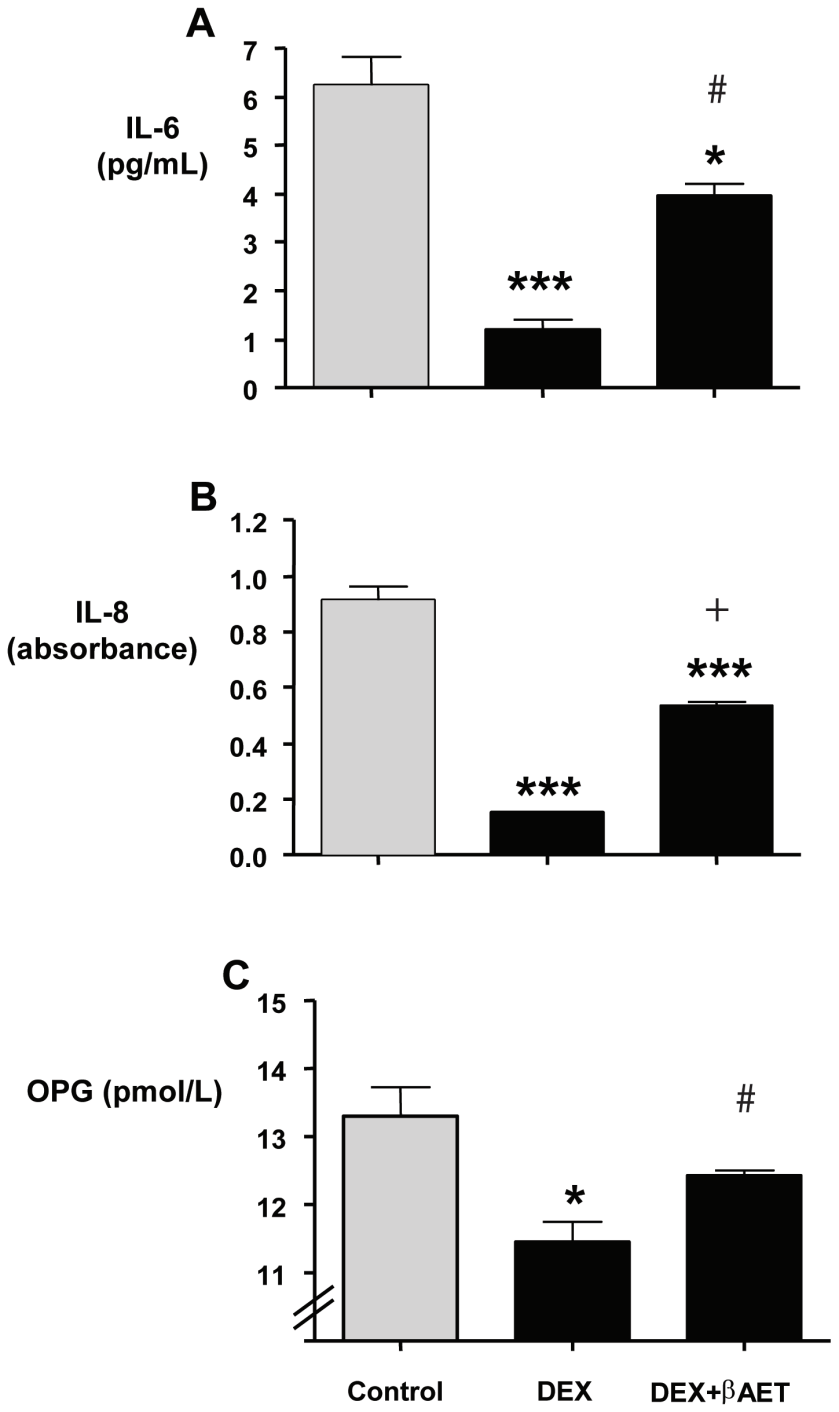
Effect of β-AET on GC-induced suppression of genes in MG-63 cell line. MG-63 cells pre-treated (1 h) with β-AET were exposed to Dex for 8 hours. The expression of IL-6 (A), IL-8 (B) and OPG (C) was determined by ELISA in the culture medium. Significant differences from control (0.04% DMSO) are indicated as follows: *** = (*p*<0.001), ** = (*p*<0.05) and * = (*p*<0.01). # indicates a significant difference from Dex (*p*<0.05). + indicates strong trend (*p*<0.1). Data are expressed as means ± sem; n = 3. Absorbance values are plotted for IL-8 (panel B) because IL-8 concentrations extrapolated from standard curve in Dex only samples were below the limits of the standard curve and could not be reliably calculated.

Endogenous GC signaling is essential for osteoblast lineage commitment, skeletal development, and normal cortical and cancellous bone volume and architecture [38], [39], [40], [41], [42]. However, disregulated levels or pharmacological GC use promotes osteopenia and has multiple effects on osteoblasts including decreased proliferation, increased apoptosis, lowered osteoprotegerin (OPN), IL-6, IL-8, and osteocalcin (OCN) expression and elevated 11βhydroxysteroid dehydrogenase-1 (11βHSD-1) expression, among others [8]. The direct effects of β-AET were explored on the human osteoblast-like MG-63 cell line. Fifty nanomolar Dex inhibited the expression of IL-6, IL-8 and OPG, an effect antagonised by 10 nM β-AET (Figure 1A-C). This amelioration reached statistical significance (*p*<0.05) for IL-6 and OPG with a strong trend (*p* = 0.057) for IL-8.

### β -AET promotes differentiation of Human MSC

**Figure 2:**
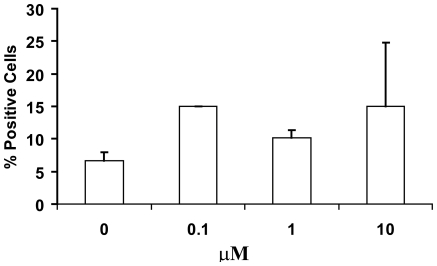
Effect of β-AET on human MSC differentiation. Mesenchymal stem cells (MSC) derived from human bone were cultured with β-AET (0, 0.1, 1 or 10 µM) for 15 days. The percentage of cells expressing the preosteoblast integrins osteopontin (OP) were measured by flow cytometry. Data are expressed as average % cells +/− standard deviation.

Mesenchymal stems cells prepared as previously described [36] and treated with β-AET showed an average of 100–200% increase in the number of cells bearing the osteopontin (Figure 2) but not the osteonectin marker (data not shown). There was no dose response as it appeared as if the lowest dose tested gave the maximum effect. The mean fluorescence intensity, reflecting the copy number per cell, was diminished modestly at the lowest 0.1 µM β-AET concentration but neither of the higher doses tested showed an effect on this parameter (data not shown).

### β-AET prevents weight loss in thermal trauma model

Mouse thermal injury models have been used to demonstrate GC and cytokine associated changes after thermal injury. Skeletal changes after burn injury have been proposed to be influenced by a number of factors such as immobilization, aluminum holding and the production of GC. The precise contribution of these and other factors to burn induced bone changes remains to be elucidated [34]. β-AET was tested for effects on bone preservation in the thermal trauma model by studying various osteogenic and physiological markers. At the end of the four-week experimental period, decreased body weight gain was observed in the mice subjected to thermal trauma compared to sham controls (2.2±0.2 g in ‘burn group’ *vs*. 2.7±0.2 g in ‘sham’ group, *p*<0.05). A similar reduction in net weight gain was observed in the low dose (25 mg/kg) treatment group while the high dose group (50 mg/kg) was statistically indistinguishable from sham (1.8 +/− 0.2 and 2.3 +/− 0.3 grams respectively; data not shown). Lean or fat body mass composition alterations were similar across all groups and differences were not significant (data not shown). These observations suggested that effects of thermal stress on global physiology and growth were minor [35].

### β-AET preserves bone mineral content, but not mineral density, from thermal trauma-induced bone loss

**Figure 3:**
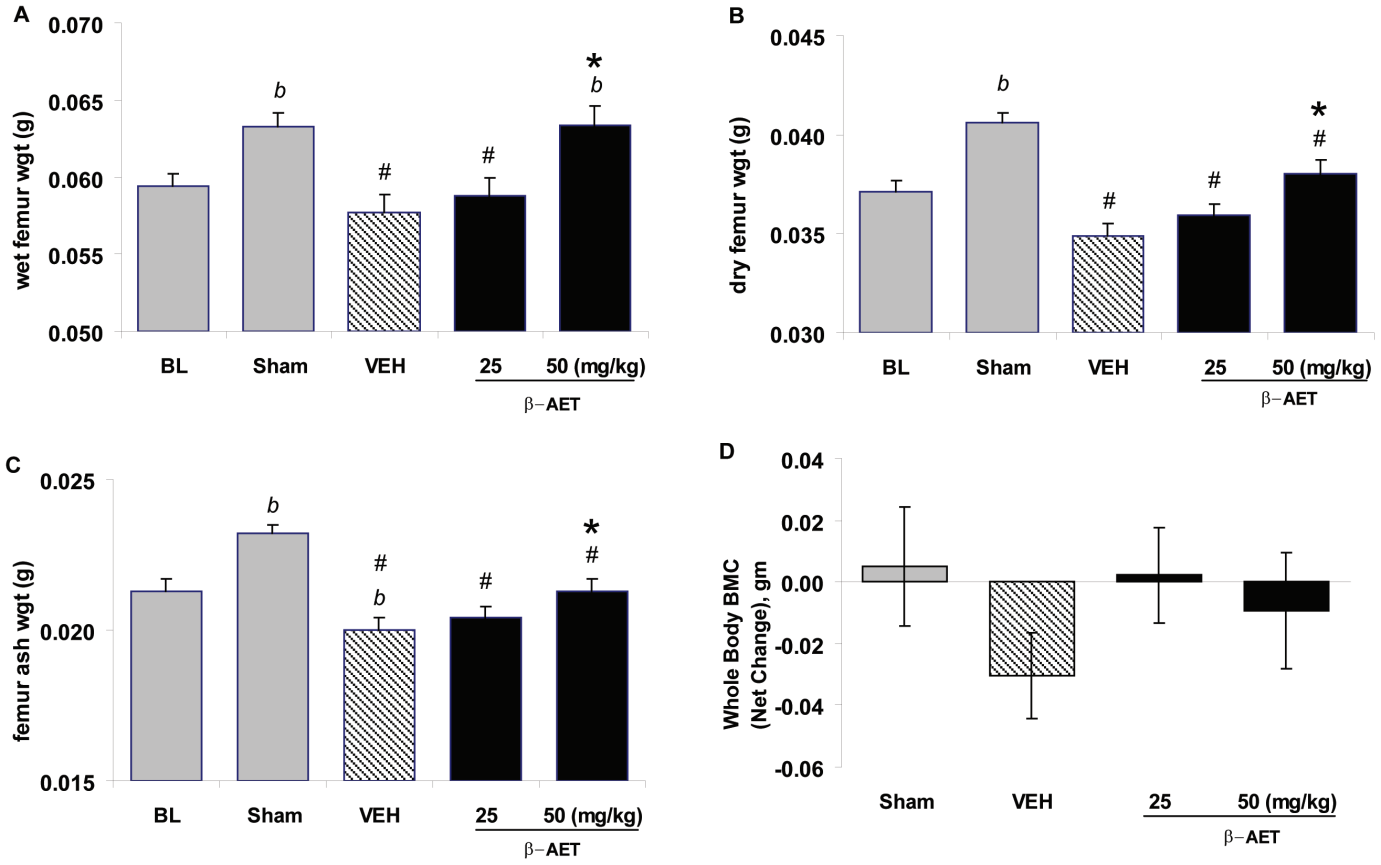
Effect of β-AET on femur weights of mice subjected to thermal injury. Male BALB/c mice (n = 10 per group) were subjected to 20% total body surface area and treated (sc injection) with vehicle alone, or with β-AET (25 or 50 mg/kg) immediately after thermal trauma. An identical treatment was given 48 hours later and then 3 times per week for 4 weeks. Femur was weighed when wet (A), after drying (B) and upon ashing (C). Panel D indicates whole body BMC. ‘#’, ‘b’ and ‘*’ indicate significant difference from the sham control, baseline and vehicle group, respectively, *p*<0.05. The bars represent means ± SEM.

Bone mineral content and weights provide a global indicator of bone health. The dry weight is a measure of inorganic and organic content, and ash weight indicates mineral content in the bone. The ash/dry weight ratios indicate the relative amounts of inorganic versus organic composition. The right femurs were weighed when wet and after drying with acetone and also after ashing. As indicated in Figure 3 (A–C), the wet, dry and ash weights, respectively, were all decreased by thermal trauma (*p*<0.05 ‘sham’ *vs*. vehicle control). These absolute weights were higher in ‘sham’ group compared to baseline as expected from normal growth during the one-month experimental period (‘baseline’ vs. ‘sham’, *p*<0.05). β-AET at the 50 mg/kg dose opposed the thermal trauma-induced attenuation of normal bone growth and mineralization as indicated by wet, dry and ash weights of the femur bone (*p*<0.05). Interestingly, the preservation of wet bone weight in 50 mg/kg group was almost complete (*p*<0.05 *vs*. vehicle) and was indistinguishable from the ‘sham’ group (Fig. 3A). Femur dry and ash weights were lagging in β-AET-treated groups compared to the ‘sham’ group, although the 50 mg/kg group was significantly different than vehicle control (*p*<0.05; Fig. 3B,C). While these results suggest a benefit of β-AET treatment on the organic component of the bone in this model, we did not observe significant differences in the ash/dry weight ratio between different groups (data not shown).

We also analyzed bone mineral content (BMC) and determined bone mineral density (BMD) at various sites. As expected, the bone mineral content and density decreased globally in thermal trauma groups (whole body measurement) and at all sites (spine, tibia and hind-limbs) tested (data not shown). BMD loss was not opposed by β-AET. However, effects from 25 mg and 50 mg/kg β-AET were similar and appeared to spare BMC-loss globally (Fig. 3D). In summary, thermal trauma decreased BMC, wet, dry and ash weights, and β-AET treatment was able to attenuate this loss.

### β-AET limits cortical cancellous bone loss and preserves endochondral growth rate but not cortical bone loss

The bones of the skeletal system are broadly classified as cortical or cancellous bones. Long bones, such as femur and tibia, consist of outer cortical bone that provides mechanical strength and an inner marrow space. The histomorphometric analysis of the femur mid-diaphysis region revealed that thermal trauma decreased the cortical area 2.6% when compared to sham, and the average and minimum cortical widths by 7.5% and 10% respectively; accordingly, the marrow space was increased by 6.4% compared to sham (data not shown). β-AET did not significantly oppose these changes in cortical bone loss (data not shown). The tibiofibular junction had a similar result.

**Figure 4:**
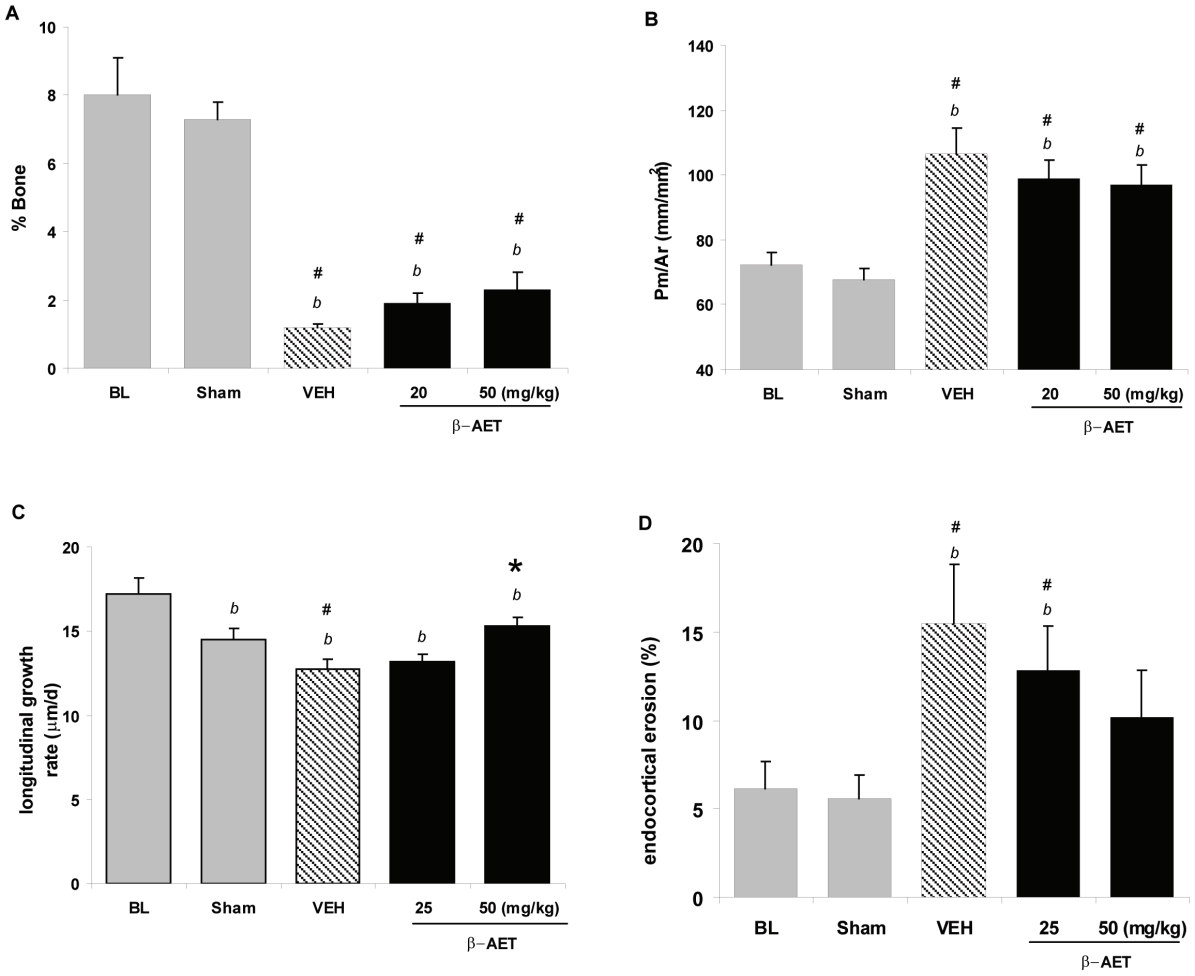
Effect of β-AET on cancellous bone morphometry and histomorphometry and bone resorption and growth. Male BALB/c mice (n = 10 per group) were subjected to 20% total body surface area burn and treated (sc injection) with vehicle alone, or with β-AET (25 or 50 mg/kg) immediately after thermal trauma. An identical treatment was given 48 hours later and then 3 times per week for 4 weeks. Cancellous bone morphometry and histomorphometry were measured at the proximal tibial metaphysis of mice (A, B). The endochondral growth rate was measured at the tibial epiphyseal growth plate (C) Endocortical eroded surface was measured at the surface of the mid-diaphyseal shaft of the femur (D), as an indicator of osteoclastic bone. ‘#’, ‘b’ and ‘*’ indicate significant differences from the sham control, baseline and vehicle group, respectively, *p*<0.05. Data are expressed as means ± SEM.

In contrast to the long bones, the flat bones (such as ribs or skull) derive mechanical strength from the trabecular fibers. Trabecular thickness, number and separation contribute to the overall quality and strength of cancellous bone. A thicker trabeculae is indicated by a lower perimeter/area ratio and, thus, increased overall cancellous bone volume and increased bone strength. The proximal ends of long bones, unlike the mid-shaft regions, are also primarily cancellous in nature. The proximal tibial metaphysis region was analyzed for effects of thermal trauma and β-AET treatment. Thermal trauma decreased cancellous bone volume by over 80% (*p*<0.05, vehicle *vs.* ‘sham’; Fig. 4A). Similarly, the effect of thermal injury was significant on trabecular integrity in this region as shown by higher perimeter/area ratios (*p*<0.05, vehicle *vs.* ‘sham’; Fig. 4B). This is consistent with the known sensitivity of this dynamic to glucocorticoid stress and constant remodeling of the bone. The β-AET treatment groups increased percent bone with a strong trend, as well as lowered the perimeter/area ratio, but without reaching statistical significance (vehicle *vs.* β-AET groups). However, these various β-AET effects did culminate into preservation of the endochondral growth rate as measured at the tibial epiphyseal growth plate, which was significantly higher in the 50 mg/kg β-AET dose group (*p*<0.05, *vs.* vehicle; Fig. 4C). The observation is consistent with β-AET opposition to GC-induced suppression of chondrocyte proliferation [43]. Since chondrogenesis is responsible for forming a template upon which osteoblasts deposit bone [44], [45], the GC-induced suppression and the integrity of the proximal tibial epiphyseal growth plate by thermal trauma may have been alleviated by β-AET.

### β-AET reduces endocortical surface bone erosion

To assess osteoclast-mediated bone erosion, we measured endocortical surface erosion. The thermal trauma increased erosion by over two-fold at the mid-diaphyseal region of femur (∼6% to 15%, ‘sham *vs*. ‘vehicle’ control, *p*<0.05) and β-AET prevented bone erosion (Figure 4D). This observation is consistent with attenuation of the glucocorticoid-mediated osteoclast activation by β-AET.

### Levels of β-AET in human plasma decrease with age

**Figure 5:**
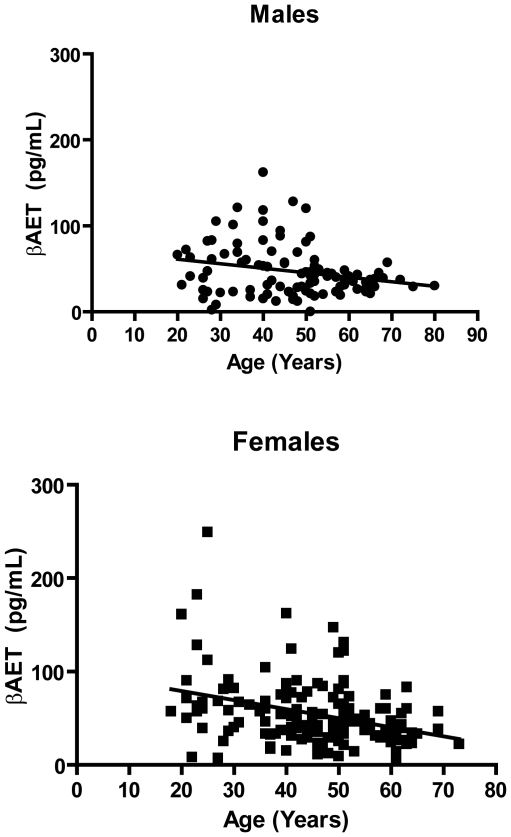
Correlation of β-AET levels in human plasma with age. β-AET levels were measured in plasma samples taken from 102 males (aged 20–80) and 150 females (aged 20–73) by reverse phase LC-MS/MS. Linear regression analysis revealed significantly non-zero slope for males (*p* = 0.02; r^2^ = 0.06) and females (*p*<0.0001; r^2^ = 0.12).

The concentration of β-AET in human plasma ranged from 2 to 162 pg/mL in males (Figure 5 Top) and from 6 to 249 pg/mL in females in our study (Figure 5 Bottom). Levels of β-AET in human plasma decrease with age. Linear regression analysis revealed significant non-zero slopes for males (*p* = 0.02; r^2^ = 0.06) and females (*p*<0.0001; r^2^ = 0.12). There was no significant difference in age between males (46.5 +/− 13.6) and females (45.6 +/− 12.5) in our sample, however, females had a significantly (*p* = 0.0002) higher BMI than males (32.0 +/− 6.4 versus 28.7 +/− 5.6, respectively). Serum levels of DHEA were available for 75 males and 68 females (data not shown). In these subjects, serum levels of DHEA also declined with age in both males and females (*p*<0.0001 for each; r^2^ = 0.21 and 0.22, respectively). As reported elsewhere (Auci *et al*., submitted) serum levels of AET were highly correlated with serum levels of DHEA in both the males (*p = *0.0008; r^2^ = 0.15) and the females (*p*<0.0001; r^2^ = 0.51). Serum levels of β-AET were also positively correlated with body mass index (BMI) and linear regression analysis of β-AET *versus* BMI revealed significant non-zero slope for males (*p* = 0.005; r^2^ = 0.076) and females (*p*<0.0001; r^2^ = 0.172).

## Discussion

We have shown that β-AET reverses GC-induced suppression of IL-6, IL-8 and OPG expression by human osteoblast-like MG-63 cells and induced osteoblast differentiation in human bone marrow derived MSC. In the mouse thermal injury model, β-AET treatment blunted the loss of bone mineralization, restored chondrocyte-mediated endochondral bone growth and slowed osteoclast-mediated cortical bone erosion. Whole body bone mineral content, but not density, was restored to normal levels. Finally, we showed that in men and women, levels of β-AET decline with age. These observations relevant to the prevention of osteopenia and may have therapeutic implications for the treatment of osteoporosis.

Osteoporosis is estimated to already affect 10 million Americans and almost 34 million more are estimated to have low bone mass, placing them at increased risk for osteoporosis. Bisphosphonates, such as Fosamax, which inhibit bone re-absorption, are commonly prescribed, despite side effects including nausea, abdominal pain, difficulty swallowing and the risk of an inflamed esophagus or esophageal ulcers. Although infrequent, serious side effects with bisphosphonates include osteonecrosis of the jaw, irregular heartbeats and visual disturbances. The drug inhibits bone-resorption and has been reported to produce brittle bones that result in an alarming frequency of spontaneous fractures [46], [47]. Selective estrogen receptor modulators (SERMs) such as Raloxifene mimic estrogen's beneficial effects on bone mineral density in postmenopausal women without the more serious side effects associated with estrogen replacement. However, these drugs also act by inhibiting bone reabsorption. The thyroid hormone calcitonin, given as a nasal spray, is less often prescribed because of its low potency compared to the bisphosphonates. Teriparatide is a powerful analog of parathyroid hormone used to treat osteoporosis in postmenopausal women and men who are at high risk of fractures. In contrast to other treatments, teriparatide acts by stimulating new bone growth. However, it must be given once a day by injection under the skin on the thigh or abdomen and the long-term effects are unknown. Thus, the development of safe, convenient and cost effective treatment options remain a significant medical and economic imperative.

The potential role of β-AET in bone formation relevant to the treatment of osteoporosis may be highlighted by our *in vitro* findings related to β-AET –induced OPG expression in Dex-treated MG-63 osteoblasts. OPG is a soluble decoy RANK receptor, secreted by osteoblasts, that binds surface RANK ligand (RANKL) on osteoblasts. This decreases the pool of RANKL available for RANK receptor binding, decreasing activation of RANK receptor on pre-osteoclasts and ultimately osteoclast commitment and differentiation [48]. Since bone formation and resorption can be uncoupled and are independent processes, the upregulation of OPG alone might tip the balance towards bone formation [49]. β-AET also attenuated GC-induced suppression of IL-6. Although high expression of IL-6 has been proposed to contribute to bone loss in GIOP, the clinical data is conflicting and it appears that the up-regulation of soluble IL-6 receptor (sIL-6Rα and its ratio to soluble gp130 co-receptor governs the effects on bone during inflammation [50], [51], [52], [53]. Local hyper-production of IL-8 has been suggested to play a specific role in subchondral bone loss, since it suppresses osteoblast activity and promotes osteoclast recruitment [54]. While not reaching statistical significance in this study, β-AET opposed GC-induced attenuation of IL-8 signaling.

The present studies are in agreement with recent work of Urban *et al*., that also suggested a beneficial effect of -AET on bone formation. Those studies used the immortalized human fetal osteoblast cell line hFOB-9 [55] and showed that β-AET suppressed PPARγ activation. PPARγ is essential for adipogenesis, endocrine function of adipose tissue and stem cell fate decisions that lead to the adipogenic commitment at the expense of the osteoblastic lineage [56]. The activation of PPARγ, as in diabetic patients managed with the thioglitazones, leads to higher incidence of bone loss. Conversely, PPARγ suppression shifts mesenchymal stem cell differentiation towards the osteoblastic lineage that supports bone formation. [56], [57], [58], [59], [60], [61], [62].

Our findings extend observations of Loria and colleagues [31], [63] to cells other than lymphocytes and macrophages and suggest β-AET attenuates the effects of GC in bone physiology. Mice that received a 20% TBSA full thickness scald injury (equivalent to third degree burn) exhibited dramatically increased bone erosion mediated by high osteoclast activity, IL-6 and GC levels, that leads to lower cancellous bone formation parameters 4–7 days later [34], [35]. Significant short-term osteoclast-mediated bone surface erosion was prevented by β-AET in a dose-dependent manner. In a feed-forward mechanism, the endocrine and cytokine signals initiated by thermal trauma may converge at the level of the hypothalamic-pitutary-adrenal axis and amplify both direct and indirect GC effects on the bone. These may include the disruption of normal osteoimmunological interactions that involve osteoblasts as well as marrow stromal cells (reviewed in [64]). Functional GC receptors have been reported on bone chondrocytes, mononuclear cells and endothelial cells, suggesting they may also be involved [65]. Even though β-AET protected BMC, it showed no effect on BMD after one month. Other factors, besides stress-induced increases in GC contribute to bone loss in thermally injured mice [34]. Our studies do not prove any interaction between β-AET and GC signaling and do not rule out other actions of β-AET relevant to the bone sparing activity we observed *in vivo*.

These observations are very similar to the bone loss and recovery dynamics in severely burned children [64]. The management of these patients with anabolic agents, recombinant growth hormone or oxandrolone, a synthetic testosterone analogue, results in a significant increase in total body or lumbar spine BMC (T-BMC or LS-BMC, respectively), but no increase in LS- spine BMD. The increase in BMC but not BMD implies a proportionate increase in bone area in patients treated with these anabolic agents. The bones become bigger, even though the mineralization per unit area, and therefore density, does not differ between treated and untreated children. Similarly, bisphosphonate palmidronate given within 10 days of burn significantly protected BMC starting within 6-8 weeks, but LS-BMD continued a downward trend over one year and Z-scores (the number of standard deviations a patient's BMD differs from the average BMD of their age, sex, and ethnicity) became significant only at 24 months [66]. The LS-BMD observation is consistent with the resumption of bone formation only after 9-12 months from the time of injury. Thus, in order to observe any significant effects on BMD in the thermal trauma mouse model, a longer follow-up period would be required.

The mechanism(s) through which β-AET exerts its pharmacological activity is presently unknown. Because β-AET is a DHEA metabolite, it is important to distinguish potential differences between the modes of action of these two molecules. Although not a PPAR ligand, DHEA stimulates PPARγ in rodents [67], and PPAR activation has a number of beneficial effects in rodent models of inflammation and metabolic disease [68]. In contrast, β-AET does not stimulate PPAR either in rodents (unpublished observations) or in humans [69]. A synthetic analog of β-AET, 17α-ethynyl-androst-5-ene-3β,7β,17β-triol (a. k. a. HE3286), has been reported to elicit rapid non-genomic effects consistent with activation of a cell membrane receptor [69]. We speculate that β-AET interacts with the same putative receptor, noting that cell surface receptors that mediate rapid non-genomic effects have also been reported for DHEA [70], progesterone, [71] estrogen [72] and androgen [73]. The ability of β-AET to modulate certain GC activities may also involve direct or indirect action on steroidogenic enzymes [74], [75] and nuclear transcription factors [76]. Since inflammation itself may exacerbate bone loss[77], a component of the benefit of β-AET in the thermal injury model may relate to anti-inflammatory properties [26].

Our finding that levels of β-AET decline with age, in the context of its role in attenuating certain aspects of GC function, may have important implications in terms of stem cell biology, osteoporosis and aging. GCs act to promote the differentiation of MSC, specifically, but perhaps not exclusively, toward the adipocyte lineage (for review see [78]). Our results suggest that the role of β-AET to modify GC activity may extend to stem cells by perhaps influencing fate decisions [79]. The specific role of β-AET may require a precise understanding of the niche [80]. Our finding that plasma levels of β-AET decrease with age make it tempting to speculate that a reduced β-AET/GC ratio is associated with an age-related decrease in tissue plasticity as it relates to stem cell activity and fate decisions. For example, the case in point, where decreasing levels of β-AET and rising levels of GC may drive MSC differentiation towards adipocyte formation at the expense of osteoblastogensis, causing an decreased bone apposition rate that leads to development of osteoporosis [78].

Whereas native steroid hormones in the C-19 steroid series such as testosterone appear to require parenteral administration to effectively observe their pharmacological activity, chemical modifications have been effective at producing forms for oral administration. HE3286 is an orally bioavailable analogue of β-AET that lacks estrogenic or androgenic side effects and has demonstrated anti-inflammatory, bone sparing properties in rodents (Harbor Biosciences, unpublished observations). HE3286 was found to be safe and tolerable in early human clinical trials. This synthetic analogue maybe an effective agent to induce GC homeostasis and safely, conveniently and cost effectively treat or prevent diseases of advancing age such as osteoporosis.

## References

[1] Webb SJ, Geoghegan TE, Prough RA, Michael Miller KK (2006). The biological actions of dehydroepiandrosterone involves multiple receptors.. Drug Metab Rev.

[2] Svec F, Porter JR (1998). The actions of exogenous dehydroepiandrosterone in experimental animals and humans.. Proc Soc Exp Biol Med.

[3] Auci DL, Ahlem C, Li M, Trauger R, Dowding C (2003). The immunobiology and therapeutic potential of androstene hormones and their synthetic derivatives: novel anti-inflammatory and immune regulating steroid hormones.. Mod Asp Immunobiol.

[4] Cleary MP (1991). The antiobesity effect of dehydroepiandrosterone in rats.. Proc Soc Exp Biol Med.

[5] Araneo BA, Woods ML2nd Daynes RA (1993). Reversal of the immunosenescent phenotype by dehydroepiandrosterone: hormone treatment provides an adjuvant effect on the immunization of aged mice with recombinant hepatitis B surface antigen.. J Infect Dis.

[6] Canning MO, Grotenhuis K, de Wit HJ, Drexhage HA (2000). Opposing effects of dehydroepiandrosterone and dexamethasone on the generation of monocyte-derived dendritic cells [In Process Citation].. Eur J Endocrinol.

[7] Clerici M, Trabattoni D, Piconi S, Fusi ML, Ruzzante S (1997). A possible role for the cortisol/anticortisols imbalance in the progression of human immunodeficiency virus.. Psychoneuroendocrinology.

[8] Harding G, Mak YT, Evans B, Cheung J, Macdonald D (2006). The effects of dexamethasone and dehydroepiandrosterone (DHEA) on cytokines and receptor expression in a human osteoblastic cell line: Potential steroid-sparing role for DHEA.. Cytokine.

[9] Wang L, Wang YD, Wang WJ, Zhu Y, Li DJ (2007). Dehydroepiandrosterone improves murine osteoblast growth and bone tissue morphometry via mitogen-activated protein kinase signaling pathway independent of either androgen receptor or estrogen receptor.. J Mol Endocrinol.

[10] Bovenberg SA, van Uum SH, Hermus AR (2005). Dehydroepiandrosterone administration in humans: evidence based?. Neth J Med.

[11] Hartkamp A, Geenen R, Godaert GL, Bijl M, Bijlsma JW (2004). The effect of dehydroepiandrosterone on lumbar spine bone mineral density in patients with quiescent systemic lupus erythematosus.. Arthritis Rheum.

[12] Sanchez-Guerrero J, Fragoso-Loyo HE, Neuwelt CM, Wallace DJ, Ginzler EM (2008). Effects of prasterone on bone mineral density in women with active systemic lupus erythematosus receiving chronic glucocorticoid therapy.. J Rheumatol.

[13] von Muhlen D, Laughlin GA, Kritz-Silverstein D, Bergstrom J, Bettencourt R (2008). Effect of dehydroepiandrosterone supplementation on bone mineral density, bone markers, and body composition in older adults: the DAWN trial.. Osteoporos Int.

[14] Jankowski CM, Gozansky WS, Schwartz RS, Dahl DJ, Kittelson JM (2006). Effects of dehydroepiandrosterone replacement therapy on bone mineral density in older adults: a randomized, controlled trial.. J Clin Endocrinol Metab.

[15] Labrie F, Luu-The V, Belanger A, Lin SX, Simard J (2005). Is dehydroepiandrosterone a hormone?. J Endocrinol.

[16] Takayanagi R, Goto K, Suzuki S, Tanaka S, Shimoda S (2002). Dehydroepiandrosterone (DHEA) as a possible source for estrogen formation in bone cells: correlation between bone mineral density and serum DHEA-sulfate concentration in postmenopausal women, and the presence of aromatase to be enhanced by 1,25-dihydroxyvitamin D3 in human osteoblasts.. Mech Ageing Dev.

[17] Wolf OT, Kirschbaum C (1999). Actions of dehydroepiandrosterone and its sulfate in the central nervous system: effects on cognition and emotion in animals and humans.. Brain Res Brain Res Rev.

[18] Marwah A, Marwah P, Lardy H (2002). Ergosteroids. VI. Metabolism of dehydroepiandrosterone by rat liver in vitro: a liquid chromatographic-mass spectrometric study.. J Chromatogr B Biomed Sci Appl.

[19] Hammer F, Subtil S, Lux P, Maser-Gluth C, Stewart PM (2005). No evidence for hepatic conversion of dehydroepiandrosterone (DHEA) sulfate to DHEA: in vivo and in vitro studies.. J Clin Endocrinol Metab.

[20] Lardy H, Marwah A, Marwah P (2002). Transformations of DHEA and its metabolites by rat liver.. Lipids.

[21] Lardy H, Marwah A, Marwah P (2005). C(19)-5-ene Steroids in Nature.. Vitam Horm.

[22] Cui H, Lin SY, Belsham DD (2003). Evidence that dehydroepiandrosterone, DHEA, directly inhibits GnRH gene expression in GT1-7 hypothalamic neurons.. Mol Cell Endocrinol.

[23] Lardy H (2003). Happily at work.. J Biol Chem.

[24] Rose KA, Stapleton G, Dott K, Kieny MP, Best R (1997). Cyp7b, a novel brain cytochrome P450, catalyzes the synthesis of neurosteroids 7alpha-hydroxy dehydroepiandrosterone and 7alpha-hydroxy pregnenolone.. Proc Natl Acad Sci U S A.

[25] Martin C, Bean R, Rose K, Habib F, Seckl J (2001). cyp7b1 catalyses the 7alpha-hydroxylation of dehydroepiandrosterone and 25-hydroxycholesterol in rat prostate.. Biochem J.

[26] Auci DL, Reading CL, Frincke JM (2009). 7-Hydroxy androstene steroids and a novel synthetic analogue with reduced side effects as a potential agent to treat autoimmune diseases.. Autoimmun Rev.

[27] Davidson M, Marwah A, Sawchuk RJ, Maki K, Marwah P (2000). Safety and pharmacokinetic study with escalating doses of 3-acetyl-7- oxo-dehydroepiandrosterone in healthy male volunteers [In Process Citation].. Clin Invest Med.

[28] Davidson MH, Weeks CE, Lardy H, Maki K, Umporowicz D (1998). Safety and endocrine effects of 3-acetyl-7-oxo DHEA (7-Keto-DHEA). FASEB Journal;.

[29] Loria RM (1997). Antiglucocorticoid function of androstenetriol.. Psychoneuroendocrinology.

[30] Padgett DA, Loria RM (1994). In vitro potentiation of lymphocyte activation by dehydroepiandrosterone, androstenediol, and androstenetriol.. J Immunol.

[31] Padgett DA, Loria RM (1998). Endocrine regulation of murine macrophage function: effects of dehydroepiandrosterone, androstenediol, and androstenetriol.. J Neuroimmunol.

[32] Padgett DA, Sheridan JF, Loria RM (1995). Steroid hormone regulation of a polyclonal TH2 immune response.. Ann N Y Acad Sci.

[33] Bowman BM, Hooper CC, Shelby J, Miller SC (2001). Comparison of an injury stress-response on skeletal growth in two inbred strains of mice;.

[34] Edelman LS, Shao W, Miller S, Bowman B, Morris SE (1997). The 1997 Lindberg Award. Effects of burn injury on bone and growth in a mouse model.. J Burn Care Rehabil.

[35] Miller SC, Bowman BM, Siska CC, Shelby J (2002). Effects of thermal injury on skeletal metabolism in two strains of mice.. Calcif Tissue Int.

[36] Reyes M, Verfaillie CM (2001). Characterization of multipotent adult progenitor cells, a subpopulation of mesenchymal stem cells.. Ann N Y Acad Sci.

[37] Bruder SP, Jaiswal N, Haynesworth SE (1997). Growth kinetics, self-renewal, and the osteogenic potential of purified human mesenchymal stem cells during extensive subcultivation and following cryopreservation.. J Cell Biochem.

[38] Eijken M, Hewison M, Cooper MS, de Jong FH, Chiba H (2005). 11beta-Hydroxysteroid dehydrogenase expression and glucocorticoid synthesis are directed by a molecular switch during osteoblast differentiation.. Mol Endocrinol.

[39] Kalak R, Zhou H, Street J, Day RE, Modzelewski JR (2009). Endogenous glucocorticoid signalling in osteoblasts is necessary to maintain normal bone structure in mice.. Bone.

[40] Sher LB, Harrison JR, Adams DJ, Kream BE (2006). Impaired cortical bone acquisition and osteoblast differentiation in mice with osteoblast-targeted disruption of glucocorticoid signaling.. Calcif Tissue Int.

[41] Sher LB, Woitge HW, Adams DJ, Gronowicz GA, Krozowski Z (2004). Transgenic expression of 11beta-hydroxysteroid dehydrogenase type 2 in osteoblasts reveals an anabolic role for endogenous glucocorticoids in bone.. Endocrinology.

[42] Zhou H, Mak W, Zheng Y, Dunstan CR, Seibel MJ (2008). Osteoblasts directly control lineage commitment of mesenchymal progenitor cells through Wnt signaling.. J Biol Chem.

[43] Mushtaq T, Farquharson C, Seawright E, Ahmed SF (2002). Glucocorticoid effects on chondrogenesis, differentiation and apoptosis in the murine ATDC5 chondrocyte cell line.. J Endocrinol.

[44] Erlebacher A, Filvaroff EH, Gitelman SE, Derynck R (1995). Toward a molecular understanding of skeletal development.. Cell.

[45] Marks SC, Lundmark C, Christersson C, Wurtz T, Odgren PR (2000). Endochondral bone formation in toothless (osteopetrotic) rats: failures of chondrocyte patterning and type X collagen expression.. Int J Dev Biol.

[46] Goddard MS, Reid KR, Johnston JC, Khanuja HS (2009). Atraumatic bilateral femur fracture in long-term bisphosphonate use.. Orthopedics.

[47] Cermak K, Shumelinsky F, Alexiou J, Gebhart MJ (2009). Case Reports: Subtrochanteric Femoral Stress Fractures after Prolonged Alendronate Therapy.. Clin Orthop Relat Res.

[48] Boyce BF, Xing L (2007). Biology of RANK, RANKL, and osteoprotegerin.. Arthritis Res Ther.

[49] Corral DA, Amling M, Priemel M, Loyer E, Fuchs S (1998). Dissociation between bone resorption and bone formation in osteopenic transgenic mice.. Proc Natl Acad Sci U S A.

[50] Dovio A, Perazzolo L, Saba L, Termine A, Capobianco M (2006). High-dose glucocorticoids increase serum levels of soluble IL-6 receptor alpha and its ratio to soluble gp130: an additional mechanism for early increased bone resorption.. Eur J Endocrinol.

[51] Buxton EC, Yao W, Lane NE (2004). Changes in serum receptor activator of nuclear factor-kappaB ligand, osteoprotegerin, and interleukin-6 levels in patients with glucocorticoid-induced osteoporosis treated with human parathyroid hormone (1-34).. J Clin Endocrinol Metab.

[52] Korczowska I, Olewicz-Gawlik A, Hrycaj P, Lacki J (2003). The effect of long-term glucocorticoids on bone metabolism in systemic lupus erythematosus patients: the prevalence of its anti-inflammatory action upon bone resorption.. Yale J Biol Med.

[53] Sahin G, Ozturk C, Bagis S, Cimen OB, Erdogan C (2002). Correlation of serum cytokine levels with axial bone mineral density.. Singapore Med J.

[54] Dovio A, Sartori ML, Masera RG, Peretti L, Perotti L (2004). Effects of physiological concentrations of steroid hormones and interleukin-11 on basal and stimulated production of interleukin-8 by human osteoblast-like cells with different functional profiles.. Clin Exp Rheumatol.

[55] Urban NH, Chamberlin B, Ramage S, Roberts Z, Loria RM (2008). Effects of alpha/beta-androstenediol immune regulating hormones on bone remodeling and apoptosis in osteoblasts.. J Steroid Biochem Mol Biol.

[56] Gunther T, Schule R (2007). Fat or bone? A non-canonical decision.. Nat Cell Biol.

[57] Kahn SE, Zinman B, Lachin JM, Haffner SM, Herman WH (2008). Rosiglitazone-associated fractures in type 2 diabetes: an Analysis from A Diabetes Outcome Progression Trial (ADOPT).. Diabetes Care.

[58] McDonough AK, Rosenthal RS, Cao X, Saag KG (2008). The effect of thiazolidinediones on BMD and osteoporosis.. Nat Clin Pract Endocrinol Metab.

[59] Meier C, Kraenzlin ME, Bodmer M, Jick SS, Jick H (2008). Use of thiazolidinediones and fracture risk.. Arch Intern Med.

[60] Yaturu S, Bryant B, Jain SK (2007). Thiazolidinedione treatment decreases bone mineral density in type 2 diabetic men.. Diabetes Care.

[61] Cock TA, Back J, Elefteriou F, Karsenty G, Kastner P (2004). Enhanced bone formation in lipodystrophic PPARgamma(hyp/hyp) mice relocates haematopoiesis to the spleen.. EMBO Rep.

[62] Akune T, Ohba S, Kamekura S, Yamaguchi M, Chung UI (2004). PPARgamma insufficiency enhances osteogenesis through osteoblast formation from bone marrow progenitors.. J Clin Invest.

[63] Loria RM, Padgett DA, Huynh PN (1996). Regulation of the immune response by dehydroepiandrosterone and its metabolites.. J Endocrinol.

[64] Klein GL (2006). Burn-induced bone loss: importance, mechanisms, and management.. J Burns Wounds.

[65] Abu EO, Horner A, Kusec V, Triffitt JT, Compston JE (2000). The localization of the functional glucocorticoid receptor alpha in human bone.. J Clin Endocrinol Metab.

[66] Przkora R, Herndon DN, Sherrard DJ, Chinkes DL, Klein GL (2007). Pamidronate preserves bone mass for at least 2 years following acute administration for pediatric burn injury.. Bone.

[67] Tamasi V, Miller KK, Ripp SL, Vila E, Geoghagen TE (2008). Modulation of receptor phosphorylation contributes to activation of peroxisome proliferator activated receptor alpha by dehydroepiandrosterone and other peroxisome proliferators.. Mol Pharmacol.

[68] Daynes RA, Jones DC (2002). Emerging roles of ppars in inflammation and immunity.. Nat Rev Immunol.

[69] Wang T, Villegas S, Huang Y, White SK, Ahlem C Amelioration of Glucose Intolerance by the Synthetic Androstene HE3286: Link to Inflammatory Pathways.. J Pharmacol Exp Ther.

[70] Liu D, Dillon JS (2004). Dehydroepiandrosterone stimulates nitric oxide release in vascular endothelial cells: evidence for a cell surface receptor.. Steroids.

[71] Vicent GP, Ballare C, Nacht AS, Clausell J, Subtil-Rodriguez A (2008). Convergence on chromatin of non-genomic and genomic pathways of hormone signaling.. J Steroid Biochem Mol Biol.

[72] Benten WP, Stephan C, Lieberherr M, Wunderlich F (2001). Estradiol signaling via sequestrable surface receptors.. Endocrinology.

[73] Foradori CD, Weiser MJ, Handa RJ (2008). Non-genomic actions of androgens.. Front Neuroendocrinol.

[74] Stewart PM, Mason JI (1995). Cortisol to cortisone: glucocorticoid to mineralocorticoid.. Steroids.

[75] Nashev LG, Chandsawangbhuwana C, Balazs Z, Atanasov AG, Dick B (2007). Hexose-6-phosphate dehydrogenase modulates 11beta-hydroxysteroid dehydrogenase type 1-dependent metabolism of 7-keto- and 7beta-hydroxy-neurosteroids.. PLoS ONE.

[76] Balazs Z, Schweizer RA, Frey FJ, Rohner-Jeanrenaud F, Odermatt A (2008). DHEA induces 11 -HSD2 by acting on CCAAT/enhancer-binding proteins.. J Am Soc Nephrol.

[77] Kong YY, Feige U, Sarosi I, Bolon B, Tafuri A (1999). Activated T cells regulate bone loss and joint destruction in adjuvant arthritis through osteoprotegerin ligand.. Nature.

[78] Feldman BJ (2009). Glucocorticoids influence on mesenchymal stem cells and implications for metabolic disease.. Pediatr Res.

[79] Kolf CM, Cho E, Tuan RS (2007). Mesenchymal stromal cells. Biology of adult mesenchymal stem cells: regulation of niche, self-renewal and differentiation.. Arthritis Res Ther.

[80] Croisille L, Auffray I, Katz A, Izac B, Vainchenker W (1994). Hydrocortisone differentially affects the ability of murine stromal cells and human marrow-derived adherent cells to promote the differentiation of CD34++/CD38- long-term culture-initiating cells.. Blood.

